# Effects of physical exercise training on nocturnal symptoms in asthma: Systematic review

**DOI:** 10.1371/journal.pone.0204953

**Published:** 2018-10-22

**Authors:** Cristina de Oliveira Francisco, Swati Anil Bhatawadekar, Jessica Babineau, W. Darlene Reid, Azadeh Yadollahi

**Affiliations:** 1 Toronto Rehabilitation Institute, University Health Network, Toronto, Ontario, Canada; 2 Department of Physical Therapy, University of Toronto, Toronto, Ontario, Canada; 3 Institute of Biomaterials and Biomedical Engineering, University of Toronto, Toronto, Ontario, Canada; The University of Melbourne, AUSTRALIA

## Abstract

**Introduction:**

Nocturnal worsening of asthma symptoms is a common feature of asthma. Physical exercise training improves general asthma control; however, there is no evidence showing the effects of physical exercise on nocturnal asthma symptoms. Indeed, asthma patients with daytime and nighttime symptoms are physiologically different, and thus the effects of physical exercise on asthma may also be different in these two groups. The objective of this systematic review is to explore the effects of physical exercise on nocturnal asthma symptoms.

**Methods:**

Searches were conducted in MEDLINE, Embase, Cochrane Central Register of Controlled Trials, CINAHL and SPORTdiscus (last search on November 2017). Authors from studies that did not report nocturnal symptoms but used questionnaires and/or diaries were contacted for detailed information. Studies that provided results on nocturnal symptoms before and after physical activity intervention were included. Prevalence of nocturnal symptoms was calculated for each study from the percentage of study participants with nocturnal symptoms before and after intervention.

**Results:**

Eleven studies were included (5 with children and 6 with adults). The prevalence of nocturnal symptoms at baseline ranged from 0% to 63% among children and from 50–73% among adults. In children and adults with nocturnal asthma, aerobic physical exercise reduced the prevalence and frequency of nocturnal symptoms.

**Conclusions:**

Aerobic physical exercise improves nocturnal asthma in children and adults by reducing the prevalence and frequency of nocturnal symptoms. Physical exercise training could be used with conventional treatments to improve quality of life and asthma control in patients with nocturnal worsening of asthma.

## Introduction

Asthma is a chronic inflammatory lung disorder characterized by variable and reversible airway obstruction and airway hyper-responsiveness [1, 2]. It is associated with recurrent episodes of wheezing, breathlessness, chest tightness and coughing [3]. Asthma affects more than 300 million people worldwide of all ages with adverse clinical, social, and economic consequences [4–6]. The financial burden on patients with asthma in different Western countries ranges from $300 to $1,300 per patient per year [7]. Moreover, asthma has a large detrimental impact on physical, emotional, social, and professional lives of sufferers [7].

Nocturnal worsening of asthma symptoms is common in more than two-thirds of asthma patients [8–11] and is associated with more severe asthma symptoms [12–15], increases in airway hyper-responsiveness, more use of asthma medications, and declines in the lung function [8, 16, 17]. Moreover, nocturnal asthma leads to fragmented sleep, daytime fatigue, and impaired cognitive performance leading to poor quality of life [16, 18, 19].

The mechanisms underlying nocturnal asthma are multifactorial [16, 17, 20, 21]. During sleep, recumbent posture causes a reduction in the lung volumes, respiratory muscle tone, and lung compliance [22–24]. Moreover, recumbent posture also causes a shift of fluid from the legs to the thorax (rostral fluid shift) that is associated with airway narrowing in asthma [25, 26]. In addition, during sleep there is an overnight decrease in plasma cortisol and an increase in airway inflammation, which in turn increases cholinergic tone [16, 17], and enhances airway hyper-responsiveness.

Evidence suggests that compared to patients without nocturnal symptoms, those with nocturnal asthma experience different physiological traits during the night [21, 27–31]. The overnight physiological abnormalities related to nocturnal asthma include: increased airway inflammation and decreased steroid responsiveness [21, 29, 30], increased pulmonary capillary blood volume [31], functional differences in blood/air volume ratios and mechanical coupling of the parenchyma to the airways [27]. Anti-inflammatory treatment and bronchodilators to relax airway smooth muscles greatly improve asthma symptoms. However, nocturnal worsening of asthma occurs even in patients under optimal medication treatment [32, 33] suggesting that pathophysiology of nocturnal asthma is not fully understood yet and complementary treatments may improve asthma control in these patients.

The beneficial effects of physical exercise on general asthma control are well documented [34–38]. Physical exercise reduces central, systemic and airway inflammation [34–37], diminishes bronchial hyper-responsiveness, and improves aerobic fitness, exercise tolerance, asthma control, and quality of life [37, 38]. Considering the physiological differences between asthma patients with daytime and nighttime symptoms, the effects of physical exercise between these two types of asthma could be distinctive. However, previous studies and systematic reviews examining the effects of physical exercise training on asthma do not specify the effects of exercise on night symptoms and sleep quality. The evidence of whether exercise exacerbates or improves nocturnal symptoms needs to be synthesized so that recommendations can be made regarding its utility in the management of patients with asthma. Therefore, the aim of this systematic review is to explore the effects of physical exercise on nocturnal asthma symptoms.

## Methods

This systematic review was conducted in accordance with Cochrane Handbook for Systematic Reviews of Intervention and Preferred Reporting Items for Systematic Reviews and Meta-Analyses (PRISMA) guidelines [39, 40]. The systematic literature review was performed using the PICO strategy (Patients: Asthmatics; Intervention: Physical Exercise; Comparisons: pre and post intervention and/or control vs intervention; Outcomes: nocturnal asthma symptoms which included episodes of wheeze or shortness of breath at night, symptoms scores specifically for nocturnal asthma symptoms, waking at night as result of asthma symptoms, scores of subjective sleep quality affected specifically by asthma symptoms, number of symptom free nights, etc.).

### Search strategy

Comprehensive searches were conducted in MEDLINE (Ovid), Embase (Ovid), Cochrane Central Register of Controlled Trials (Ovid), CINAHL (EBSCO) and SPORTdiscus (EBSCO). In addition, a supplemental search was conducted in PubMed to identify non-MEDLINE records. The searches were developed and executed by an expert reference librarian (JB). Search strategies included a combination of text words and subject headings (e.g. MeSH, Emtree) relating to: (1) physical activity or exercise (2) asthma, and (3) trials. We did not explicitly include search terms related to nocturnal symptoms or subjective sleep quality in the search strategies as these terms are rarely mentioned in the title, abstract, or subject headings, but are more often embedded within the article’s full-text and thus, must be identified at the screening level. Each database search was conducted from its respective inception until November 2017. Searches were limited to English language papers and humans. Details of the MEDLINE search strategy are outlined in supplementary file (S1 Text).

### Study selection and data extraction

Two independent reviewers (COF and SAB) performed the screening process to determine potential relevance. Our inclusion criteria were: 1) clinical trials with asthma patients 2) intervention with any type of physical exercise training lasting at least 20 minutes per day, undertaken at least two times per week for a minimum duration of four weeks (following the criteria used in the Cochrane systematic review regarding physical activity for asthma, Carson et al 2013[37]); 3) studies evaluating nocturnal asthma symptoms, or subjective sleep quality affected by asthma symptoms before and after physical exercise and 4) studies in English. Studies that did not report nocturnal symptoms, or subjective sleep quality but used instruments (questionnaires and/or diaries) that include questions about these parameters were included. In addition, authors of these studies were contacted to obtain information about the specific questions related to the overnight symptoms and subjective sleep quality before and after physical activity.

The exclusion criteria included studies that investigated: animals; subjects without asthma; asthma patients combined with subjects with other diseases; other interventions besides physical activity; only respiratory muscle training and breathing exercises; reviews, guidelines, letters, commentaries, book chapters or published only as an abstract or poster. Studies without extractable nocturnal symptoms and/or subjective sleep quality data were excluded if no further information was acquired after three attempts to contact the study authors.

For each reference, the following variables were systematically extracted and entered into a summary table: 1) authors; 2) publication year; 3) journal; 4) type of physical activity, duration, progression and frequency; 5) nocturnal symptoms assessment tool 6) sample size; 7) age, body mass index (BMI) and medication at baseline; 8) study design; 9) nocturnal symptoms; 10) general asthma symptoms 11) results related to the changes in asthma symptoms (inflammatory markers, exercise capacity, lung function, etc.). Details of the full data extracted from the papers are outlined in supplementary file (S1 Table).

Two independent reviewers (COF and SAB) assessed the risk of bias for included studies as per The Cochrane Handbook for Systematic Reviews of Intervention criteria [39].

### Nocturnal symptoms assessment tool

The studies included in the systematic review assessed nocturnal symptoms and subjective sleep quality using different methods summarized in Tables 1 [41–45] and 2 [46–51]. To evaluate asthma symptoms, seven studies used the Asthma Control Questionnaire (ACQ) [41, 45–47, 49–51]. ACQ has been validated for adults and children [15, 52] and integrates 7 questions about asthma management, including one question about the frequency of nocturnal asthma symptoms (ACQ-1 “On average, during the past week, how often were you woken by your asthma during the night?”). All ACQ questions are weighed equally and the ACQ score is the average of the responses ranging from 0 to 6 (higher ACQ score is worse asthma control). ACQ score ≤ 1.5 is considered to be well-controlled asthma [53] and reduction of ≥ 0.5 in the score is considered to be a clinically significant improvement in asthma control [54].

**Table 1:** Summary of studies characteristics with children participants.

Author, year [ref]	Comparison groups	Groups: n	Sex (F/M)	Age (yr)	Intervention	Nocturnal symptoms sources
Gomes, 2015 [41]	Physical exercise (video game)	Children with asthma: 13	4/9	7.5±1.9	40min 2x/wk for 8w of video game exercise	ACQ
	Physical Exercise (treadmill)	Children with asthma: 13	5/8	8.0±2.0	40min 2x/wk for 8w of treadmill exercise	
Haines 2013 [42]	Physical exercise (pre vs post)	Children with asthma: 10	4/6	9.3±1.5	At least 30min of 5–7 physical activities/wk for 6wk of unsupervised physical activity (aerobic and strength exercises)	Authors created questionnaire
Moreira, 2008 [43]	Physical exercise	Children with asthma: 17	6/11	12.9±3.4	50min 2x/wk for 12wk of aerobic and strength exercises	PAQLQ
	Control	Children with asthma: 17	8/9	12.5±3.5	12 wk without exercise (control)	
Weisgerber, 2003 [44]	Physical exercise	Children with asthma: 5	2/3	8.4±1.5	45min 2x/wk for 5-6wk of swim classes	Authors created questionnaire
	Control	Children with asthma: 3	2/1	7.3±0.6	4-6wk without exercise (control)	
Westergren, 2016 [45]	Physical Exercise (pre vs post)	Children with asthma: 6	2/4	10.5 (10–12)	1h 2x/wk for 6w of aerobic exercise (games)	ACQ and PAQLQ

Data are reported as mean ± SD or median (min-max).

ACQ, Asthma Control Questionnaire; PAQLQ, Pediatric Asthma Quality of Life Questionnaire; AQLQ, Asthma Quality of Life Questionnaire.

**Table 2:** Summary of studies characteristics with adults participants.

Author, year [ref]	Comparison groups	Groups: n	Sex (F/M)	Age (yr)	Intervention	Nocturnal symptoms sources
Dogra, 2011 and 2010 [46, 47]	Physical exercise (supervised + unsupervised)	Non-obese adults with asthma: 18	13/5	34.2±3.2	1h 3x/wk for 12 wk of aerobic and strength exercise + 30min 5x/wk for 12wk unsupervised exercise	ACQ and mini-AQLQ
	Physical exercise (unsupervised)	Non-obese adults with asthma: 12	9/3	31.2±4.2	30min 5x/w for 12 wk of unsupervised physical activity (aerobic and strength exercises)	
	Control	Non-obese adults with asthma: 12	9/3	34.0±3.4	12 wk without exercise (control)	
Hallstrand, 1999 [48]	Physical exercise	Non-obese adults with asthma: 5	5/0	28.8	At least 30min 3x/wk for 10wk of aerobic exercise	Symptoms diary
	Control	Healthy: 5	4/1	31	Control: 10 wk without exercise	
Scott 2013 and 2015 [49, 50]	Physical exercise	Obese adults with asthma: 10	5/5	42.2±11.5	1h 3x/wk for 10wk of aerobic and strength exercise	ACQ and AQLQ
	Physical exercise + diet	Obese adults with asthma: 13	7/6	33.9±11.5	1h 3x/wk for 10wk of aerobic and strength exercise + 10 wk of reduction in food intake	
	Diet	Obese adults with asthma: 15	8/7	44.7±14.7	10wk of reduction in food intake	
Turk 2017 [51]	Physical exercise in non-obese asthmatics	Non-obese adults with asthma: 35	19/16	41.0±10.0	1h 3x/wk for 12wk of aerobic and strength exercises	ACQ
	Physical exercise in obese asthmatics	Obese adults with asthma: 19	14/5	43±7.5		

Data are reported as mean ± SD.

ACQ, Asthma Control Questionnaire; AQLQ, Asthma Quality of Life Questionnaire.

Two studies used the Asthma Quality of Life Questionnaire (AQLQ) [49, 50] and other two used the mini-AQLQ [46, 47]. These four studies used the quality of life questionnaires in combination with ACQ. Two studies used Pediatric Asthma Quality of Life Questionnaire (PAQLQ) [43, 45]. The mini-AQLQ is a short version of AQLQ, and the PAQLQ is the version for children. All of these quality of life questionnaires are evaluated on scales of 1 to 7 and the total score is the mean of all items. A higher score indicate better quality of life [55, 56] and an increase in the score of ≥ 0.5 is considered to be a clinically significant improvement [57]. The PAQLQ and AQLQ have one question related to frequency of nocturnal symptoms (PAQLQ-16 “How often did your asthma wake you up during the night during the past week?” and AQLQ-24 “How often during the past two weeks have you been woken at night by your asthma?”) and one question about subjective sleep quality (PAQLQ-20 “How often did you have trouble sleeping at night because of your asthma during the past week?” and AQLQ-29 “How often during the past two weeks has your asthma interfered with getting a good night`s sleep?”) whereas the mini-AQLQ has only one question about subjective sleep quality (mini-AQLQ-8 “How much of the time during the last 2 weeks did you have difficulty getting a good night`s sleep as a result of your asthma?”).

Two studies used their own symptom questionnaire to report the frequency of nocturnal symptoms [42, 44]. Haines et al [42] had one question regarding the incidence of nightly asthma symptoms and Weisgerber et al [44] had one question regarding the frequency of coughs at night. Both questionnaires had one question about nocturnal symptoms evaluated on a scale of 1 to 4. Higher scores indicated more nights per week with awakening due to nocturnal symptoms. One study used a patient reported diary of daytime and nighttime symptoms [48].

The above sources used for evaluating asthma control, nocturnal asthma and subjective sleep quality data were related questionnaires for asthma population. The questions regarding subjective sleep quality are specific for sleep quality affected by asthma symptoms. Global sleep quality was not evaluated in the present study. Moreover, these questionnaires evaluate asthma control and nocturnal asthma based on the subjective impressions of the participants regarding the frequency of their symptoms. For this reason, these questionnaires may not be very sensitive to mild changes in nocturnal symptoms. Thus, we calculated the prevalence of nocturnal symptoms (percentage of participants with nocturnal symptoms) as a measurement of presence or absence of symptoms. In the studies that used ACQ, AQLQ and PAQLQ [41, 43, 45–47, 49–51], nocturnal symptoms were considered absent when the patients reported no nocturnal symptoms at all (any nocturnal awakening or nighttime symptoms). Studies that used their own symptom questionnaire [42, 44] combined the patients with no nocturnal symptoms and those with very low frequency of nocturnal symptoms. We could not reach out to the authors to confirm the exact number of participants without any nocturnal symptoms. For these studies, nocturnal symptoms were considered absent when patients scored 1 (lower frequency of night symptoms available in their scale).

### Statistical analysis

We calculated the changes in the scores for each of the respective single question regarding nocturnal symptoms and subjective sleep quality from the studies where these data were extractable [42, 44] or from the data provided by the authors [41–51]. Data are presented as mean ± SD. The normality of data was tested using the Shapiro-Wilk test. Changes in nocturnal symptoms scores and in subjective sleep quality from pre to post intervention were compared with a paired t-test or Wilcoxon signed-rank test. For the studies with more than one group, between group changes were compared using two-way ANOVA with time (pre and post intervention) and groups (exercise and control) as factors. The prevalence of nocturnal symptoms was calculated from the percentage of study participants with nocturnal symptoms pre and post intervention. Statistical significance was accepted at p < 0.05. All statistical analyses were performed using SAS (version 9.3 Institute, Cary, NC, USA).

## Results

### Characteristics of included studies

**Fig 1:**
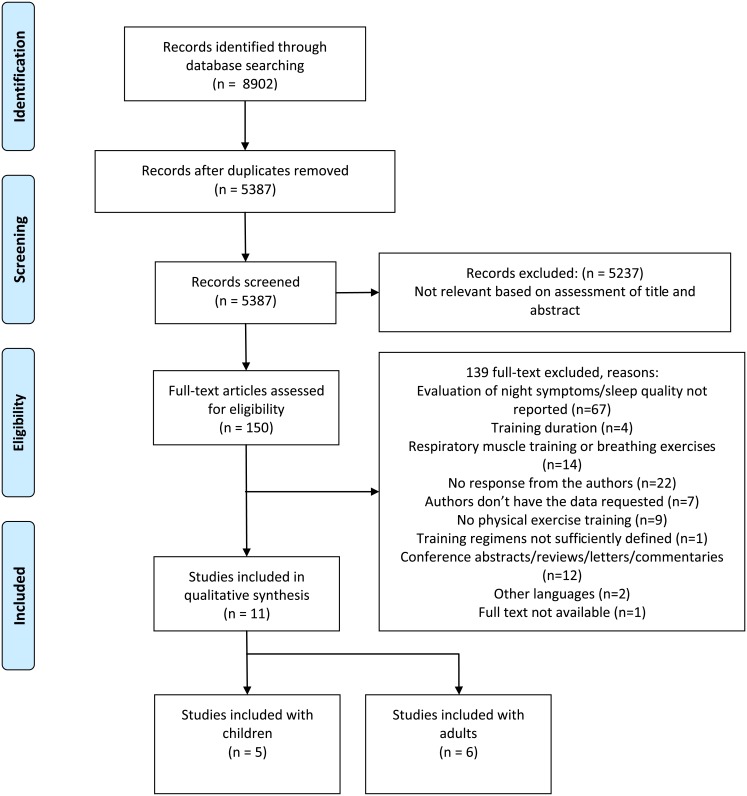
Flow diagram of the study inclusion.

A summary of the screening process is presented in Fig 1. The search resulted in 8902 titles from the initial search of the above mentioned databases. After eliminating duplicates and screening of titles and abstracts, 150 full texts were reviewed, out of which 37 were considered to be potentially relevant [41–51, 58–83]. The authors of these 37 studies [41–51, 58–83] were contacted to obtain the specific information about nocturnal symptoms and/or subjective sleep quality. Three studies reported nocturnal symptoms in the published articles [42, 44, 48] and authors of eight studies provided the specific information related to the outcomes of this systematic review [41, 43, 45–51]. Therefore, a total of 11 studies were included in this systematic review. Of these included studies, five studies evaluated children (7–15 years old) [41–45] (Table 1) while six evaluated adult participants (over the age of 18 years) [46–51] (Table 2). The detailed characteristics of the included studies with children and adults are summarized in Tables 1 and 2, respectively. In the studies of adults, two studies compared the same control group with unsupervised physical exercise training [46] and supervised physical exercise training [47] and thus, these data are presented in combination. Moreover, Scott et al. published a retrospective analysis in 2015 [50] of the same patient cohort described previously by Scott et al. in 2013 [49]. For this reason, these two reports are described together.

### Risk of bias in included studies

**Fig 2:**
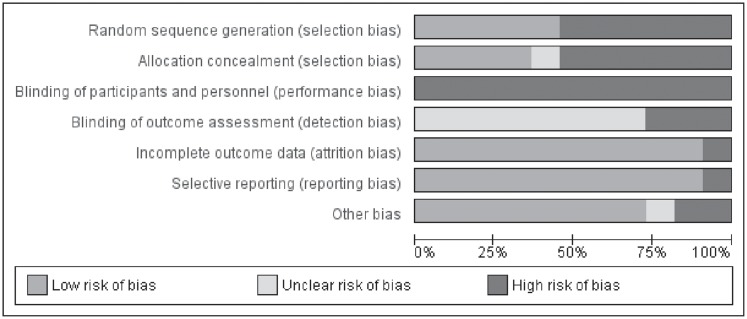
Methodological quality graph. Review authors’ judgements about each methodological quality item presented as percentages across all included studies.

The risk of bias assessment for included studies is summarized in the Fig 2. Five out 11 studies (45%) [41, 43, 44, 49, 50] were randomized control trials (RCT), with a low risk of bias for allocation. However, one of these RCTs [44] did not provide details about the allocation concealment, and thus was considered with an unclear risk of bias for this item. Due to the nature of intervention in these studies (physical exercise), blinding of participants was not possible. Additionally, blinding of outcome assessment was not performed in 3 studies [43, 49, 50] while remaining 8 studies [41, 42, 44–48, 51] had an unclear risk of bias for this item. One study [44] had evident incomplete outcome reporting data, with significant large number of dropouts. In addition, we identified a study with reporting bias [51], not presenting group comparisons for the main outcomes. Moreover, we identified other potential source of bias in 2 studies [44, 46] and an unclear other source of bias in 1 study [41]. Dogra et al [46] reported poor adherence for unsupervised exercise protocol which may be a source of bias. Weisgerber et al [44] had baseline imbalance for height and forced vital capacity between their groups which was considered as a source of bias. Finally, Gomes et al [41] had an unclear other source of bias because there was no information about adherence rate, or percentage of exercise sessions attended.

### Nocturnal symptoms and subjective sleep quality in children

Of five studies on children, [41–45] three evaluated the effects of aerobic exercise training on asthma symptoms [41, 44, 45] and two evaluated the effects of combined aerobic and strength exercise training on asthma symptoms [42, 43] (Table 1). These 5 studies with children had heterogeneous inclusion criteria. Two studies included children based on their asthma severity (moderate persistent asthma) [42, 44], two included children based on their asthma control (controlled asthma, i.e., no exacerbation or change in medication in the previous 30 days) [41, 43] and one study [45] did not use asthma severity or level of control as an inclusion criterion. In this study [45], based on the ACQ scores [15, 53], the authors included children with well-controlled or relatively well-controlled asthma.

Table 3 shows the summary of changes in the nocturnal symptoms after physical exercise in children. The prevalence of children with nocturnal symptoms at baseline ranged from 0% to 63%. The duration of the exercise training among the studies was between 5 and 8 weeks. Eighty percent of the studies with children (4 studies) [41–44] showed a reduction in the prevalence of nocturnal symptoms after physical exercise training. Only Westergren et al [45] did not find changes in the prevalence of nocturnal symptoms after physical exercise training; however, in this study there were no participants with nocturnal symptoms at baseline.

**Table 3:** Summary of effects of physical exercise training on nocturnal symptoms in children with asthma.

Author, year [ref]	Comparison groups: n	General asthma symptoms	Nocturnal symptoms prevalence and changes from pre to post intervention	Nocturnal symptoms findings
Gomes, 2015 [41]	8wk of exercise (video game): 13	Asthma control improved in both groups	Prevalence: 71% to 14% ΔACQ1: -1.4±1.4, p = 0.002	Both groups showed reduction in nocturnal symptoms frequency with no differences between groups
	8wk of exercise (treadmill): 13		Prevalence: 54% to 15%. ΔACQ1: -0.8±0.9, p = 0.02	
Haines, 2013 [42]	6wk of unsupervised exercise: 10	Physical exercise program significantly improved asthma control	Prevalence: 30% to 10% Nocturnal symptoms (authors`questionnaire): -0.2±0.4, p = 0.5	No changes in nocturnal symptoms frequency
Moreira, 2008 [43]	12wk of exercises: 17	Exercise group improved asthma-related quality of life	Prevalence: 60% to 53%. ΔPAQLQ-16: 0.1±0.7, p = 0.8 ΔPAQLQ-20: 0.1±0.8, p = 0.8	Within each group, there were no significant changes in nocturnal symptoms frequency
	Control: 17		Prevalence: 40% to 40% ΔPAQLQ-16: 0.5±1.9, p = 0.5 ΔPAQLQ-20: 0.0±1.4, p = 1.0	
Weisgerber, 2003 [44]	5-6wk of exercise: 5	No changes in asthma symptom score after swimming program	Prevalence: 80% to 60% Nocturnal symptoms (authors`questionnaire): 0.0±1.4, p = 1.0	Within each group, there were no significant changes in nocturnal symptoms frequency
	Control: 3		Prevalence: 33% to 100% Nocturnal symptoms (authors`questionnaire): 1.3±2.1, p = 0.4	
Westergren, 2016 [45]	6wk of exercise (games): 6	All children were classified as with well controlled asthma at baseline. Asthma-related quality of life improved post- exercise.	No nocturnal symptoms at baseline ΔACQ-1: -0.3±0.5, p = 0.5 ΔPAQLQ-16: -0.0±0.6, p = 1.0 ΔPAQLQ-20: -0.3±0.5, p = 0.5	No changes in nocturnal symptoms frequency and subjective sleep quality

Data are reported as mean ± SD.

ACQ-1, Asthma Control Questionnaire question about nocturnal asthma symptoms frequency; PAQLQ-16, Pediatric Asthma Quality of Life Questionnaire question about nocturnal asthma symptoms frequency; PAQLQ-20, Pediatric Asthma Quality of Life Questionnaire question about subjective sleep quality.

Three studies [42–44] found reductions in the prevalence of nocturnal symptoms after physical exercise training, but did not find significant changes in the questionnaire scores related to the frequency of nocturnal symptoms. Regarding these results, Haines et al [42] included a few participants with nocturnal symptoms at baseline (30%). Moreover, using the symptom questionnaire created by the authors, on a scale of 1 to 4, the patients without nocturnal symptoms scored 1 and the patients with nocturnal symptoms scored 2, indicating that the patients with nocturnal asthma had mild symptoms resulting in a small mean change in the score. After 5–6 weeks of swimming twice a week, Weisgerber et al [44] did not find any changes in the nocturnal symptoms scores. However, this study included only 8 children, and they had high variability in the nocturnal symptom score (Table 3). Moreira et al [43] evaluated 12 weeks of physical training (twice per week, 50 min each) in 15 children with no changes in medications throughout the study period. They did not find any improvement in nocturnal symptoms score but a small reduction in the prevalence of nocturnal asthma symptoms from 60% to 53% in the exercise group. Similar to [44], in this study, there was also a high variability in the nocturnal symptoms score in both exercise and control groups (Table 3).

Gomes et al [41] was the only study in which the children had significant improvements in the nocturnal symptoms scores. They evaluated the effects of two modalities of aerobic exercise (active video game and treadmill) for 8 weeks on asthma. There were no changes in asthma medications throughout the study period. Both exercise interventions decreased the frequency of nocturnal awakening (evaluated using ACQ). The reduction in nocturnal symptoms between the two groups was similar (p = 0.1).

### Nocturnal symptoms and subjective sleep quality in adults

Six studies evaluated the effects of physical activity on nocturnal symptoms in adults with asthma [46–51]. Five of these studies evaluated the effects of combined aerobic and strength exercise training on asthma symptoms [46, 47, 49–51] and one study evaluated the effects of aerobic exercise training on asthma symptoms [48] (Table 2). The duration of the exercise training in different studies was between 10 and 12 weeks. Moreover, these studies had a wide range of inclusion criteria regarding level of asthma control. One study included adults with partially controlled asthma (i.e. those who experienced asthma symptoms regularly, but symptoms were not severe) [46], one study included only individuals requiring only intermittent short-acting β_2_-agonists therapy 3 months preceding the study [48], and four studies did not use asthma severity or level of control as an inclusion criterion [47, 49–51] but measured patients’ asthma control using the ACQ [15, 53]. Based on the ACQ scores[15, 53], Dogra et al [47] included participants with well-controlled or relatively well-controlled asthma, Turk et al [51] included participants with relatively well-controlled or poorly controlled asthma, and Scott et al [49, 50] included participants with well-controlled, relatively well-controlled or poorly controlled asthma.

Table 4 summarizes changes in nocturnal symptoms after intervention in adults. The prevalence of nocturnal symptoms at baseline ranged from 50 to 74%. Four of six studies with adults [47, 49–51] showed a reduction in the prevalence of nocturnal symptoms after physical exercise training.

**Table 4:** Summary of effects of physical exercise training on nocturnal symptoms in adults with asthma.

Author, year [ref]	Comparison groups: n	General asthma symptoms	Nocturnal symptoms prevalence and changes from pre to post intervention	Nocturnal symptoms findings
Dogra, 2010 and 2011 [46, 47]	12 wk of exercise: 18	Asthma quality of life and asthma control improved after supervised and unsupervised exercise training.	Prevalence: 72% to 28% ΔACQ-1: -0.5±0.9, p = 0.06 Δmini-AQLQ-8: 0.9±1.3, p = 0.007	A clinically and statistically significant improvement in subjective sleep quality and a clinically significant reduction in nocturnal symptoms frequency were observed in the supervised exercise group Compared to the control group, in the supervised exercise group, subjective sleep quality improved and frequency of nocturnal awakening reduced
	12 wk of unsupervised exercise: 12		Prevalence: 46% to 62% ΔACQ-1: 0.3±0.8, p = 0.3 Δmini-AQLQ-8: -0.2±1.0, p = 0.4	
	Control: 12		Prevalence: 58% to 50% ΔACQ-1: 0.1±0.8, p = 1.0 Δmini-AQLQ-8: 0.0±1.0, p = 1.0	
Hallstrand 1999 [48]	10 wk of exercise in asthma: 5	No changes in asthma symptom scores after exercise program	Data is not available to calculate prevalence No changes in nocturnal symptoms	There were no significant changes in the nocturnal symptoms frequency within each asthma group
	10 wk of exercise in healthy: 5		Data is not available to calculate prevalence No changes in nocturnal symptoms	
Scott 2013 and 2015 [49, 50]	10 wk of exercise in obese with asthma: 10	All three interventions improved asthma-related quality of life. Asthma control improved in diet group and combined interventions (diet+exercise)	Prevalence: 50% to 30% ΔACQ-1: -0.3±0.8 p = 0.5 ΔAQLQ-24: 0.3±1.3 p = 0.6 ΔAQLQ-29: 0.5±1.4, p = 0.3	A clinically significant improvement in subjective sleep quality was observed in exercise and exercise + diet group Within each group, there were no significant changes in the nocturnal awakening frequency
	10wk of exercise and diet in obese with asthma: 12		Prevalence: 67% to 42% ΔACQ-1: -0.5±1.1 p = 0.2 ΔAQLQ-24: 0.8±1.0, p = 0.06 ΔAQLQ-29: 0.8±0.8, p = 0.02	
	10 wk of diet in obese with asthma: 16		Prevalence: 38% to 19% ΔACQ-1: -0.1±0.7 p = 0.8 ΔAQLQ-24: 0.4±1.0, p = 0.2 ΔAQLQ-29: 0.3±1.1, p = 0.3	
Turk 2017 [51]	12wk of exercise in non-obese with asthma: 35	Asthma control was improved in both groups	Prevalence: 71% to 51% ΔACQ-1: -0.6±1.5, p = 0.03	Reduction in nocturnal awakening frequency was clinically significant in both groups, but statistically significant only in the non-obese group
	12wk of exercise in obese with asthma: 19		Prevalence: 79% to 68% ΔACQ-1: -0.6±1.7, p = 0.1	

Data are reported as mean ± SD.

ACQ-1, Asthma Control Questionnaire question about nocturnal asthma symptoms frequency; AQLQ-24, Asthma Quality of Life Questionnaire question about nocturnal asthma symptoms frequency; AQLQ-29, Asthma Quality of Life Questionnaire question about subjective sleep quality; Δmini-AQLQ-8, mini- Asthma Quality of Life Questionnaire question about subjective sleep quality.

Four studies evaluated non-obese adults with asthma [46–48, 51]. Two of these studies [47, 51] showed that supervised physical exercise for at least 12 weeks (3 times per week) reduced the nocturnal asthma symptoms prevalence, improved subjective sleep quality and reduced nocturnal symptoms frequency. The studies did not report if there were any changes in medications over the study period. Dogra et al [47] also evaluated the effects of additional 12 weeks of unsupervised physical exercise after the 12 weeks of supervised exercise training. They found that subjective sleep quality and nocturnal awakening frequency scores were maintained by unsupervised physical exercise (mean change: 0.2 ± 1.0, p = 0.7 for nocturnal symptoms frequency and -0.3 ± 1.2, p = 0.8 for subjective sleep quality). On the other hand, a similar duration of unsupervised exercise (12 weeks) which was not anteceded by supervised exercise did not provide similar results [46]. Moreover, Hallstrand et al [48] reported no changes in the daytime and nocturnal symptoms and medication after 10 weeks of aerobic exercise. However, the baseline asthma symptoms were not reported in the paper and we could not reach the authors to get this information.

Two studies investigated the effects of physical exercise training in obese patients with asthma [49–51]. These studies showed that in obese adults with asthma, physical exercise for at least 10 weeks (3 times per week) reduced the prevalence of nocturnal asthma and was associated with clinically significant improvements in nocturnal symptoms and subjective sleep quality but they did not report if there were any changes in medication over the study period. Turk et al [51] compared the effects of 12 weeks of supervised physical exercise training in obese and non-obese patients with asthma (Table 4). In this study, the awakening frequency score was available from 54 patients. From this sample, the percentage of patients with nocturnal symptoms reduced from 74% at baseline to 57% after exercise training and the prevalence of nocturnal symptoms decreased in obese and non-obese groups (Table 4). Both groups had clinically significant reductions in the nocturnal symptoms frequency (reduction of ≥ 0.5 in the score; Table 4) and the changes in nocturnal symptoms frequency were similar between the two groups. However, only in non-obese patients there was a statistically significant reduction in the scores of nocturnal symptoms frequency (p = 0.03).

Scott et al [49, 50] investigated the effects of three weight loss interventions (diet, exercise, and diet plus exercise) on clinical outcomes of asthma. All weight loss interventions improved asthma-related quality of life and reduced the prevalence of nocturnal symptoms (Table 4). Clinically significant improvements in subjective sleep quality (increase of ≥ 0.5 in the score, Table 4) were found only in the groups that performed exercise (exercise plus diet and exercise groups). However, subjective sleep quality improvement was statistically significant only in the diet plus exercise group (Table 4). In all three groups, there were no statistically significant changes in the nocturnal symptoms frequency. However, in the diet plus exercise group, reduction in the nocturnal symptoms frequency was borderline significant (p = 0.06) and also clinically significant.

## Discussion

This is the first systematic review to explore the effects of physical exercise on nocturnal asthma symptoms. We included 11 studies and our main finding is that aerobic physical exercise may improve nocturnal asthma in children and adults by reducing the prevalence and frequency of nocturnal symptoms.

In the studies with children, physical exercise for at least 6 weeks, twice a week, reduces the prevalence of nocturnal symptoms [41–44]. Moreover, supervised aerobic physical exercise for 8 weeks (2 times per week) improves nocturnal symptoms and aerobic fitness and reduces pulmonary inflammation [41]. The prevalence of children with nocturnal symptoms at baseline ranged from 0–63%. However, some studies included controlled asthma, possibly excluding patients with severe nocturnal symptoms. Thus, the heterogeneity in the inclusion criteria among the studies could contribute to the discrepancy in the prevalence of nocturnal symptoms.

In studies with non-obese adults with asthma, supervised physical exercise for at least 12 weeks reduced the nocturnal asthma prevalence, improved subjective sleep quality, and reduced nocturnal symptoms frequency [47, 51]. Unsupervised exercise training for the same period (12 weeks) did not improve nocturnal symptoms [46], but was effective to control nocturnal symptoms frequency after the supervised exercise period [47]. The improvements in general asthma control after aerobic physical exercise training were associated with the improvements in aerobic fitness and dyspnea index [47, 51]. However, based on the included studies, it is not possible to determine the mechanisms by which physical exercise can improve nocturnal asthma in non-obese patients.

In obese adults with asthma, physical exercise for at least 10 weeks reduced the prevalence of nocturnal asthma and was associated with clinically significant reduction in nocturnal symptoms and enhanced subjective sleep quality [49–51]. Scott el at [49, 50] investigated the effects of three weight loss interventions (diet, exercise, and diet plus exercise) on clinical outcomes of asthma. In this study, all interventions led to a significant weight loss in all groups. However, the authors found clinically significant improvements in subjective sleep quality only in the groups that performed physical exercise. Thus, it is not clear whether in obese patients with asthma, weight loss plays an additional role in reducing nocturnal symptoms frequency and improving asthma control. In Scott el at studies [49, 50], the diet group had a few participants with nocturnal symptoms at baseline (38% of the participants), and the symptoms were mild (mean score of subjective sleep quality at baseline of 6.4 ± 0.6 in a scale of 7).

The same study [49] showed that physical exercise reduced eosinophilic airway inflammation independent of the weight loss. On the other hand, neutrophilic airway inflammation reduction was associated with adipose tissue diminution [49]. Together, these results indicate that in obese asthma patients, the combination of eosinophilic and neutrophilic inflammation pathways may be related to the nocturnal asthma [84, 85]. Because the prevalence of nocturnal symptoms was variable between the intervention groups and we did not have enough information about inflammation or weight loss from other included studies, it was not possible to determine which inflammatory pathway may be more important in the pathophysiology of nocturnal asthma or which intervention (exercise or weight management) is more effective in reducing the prevalence of nocturnal asthma in obese patients. Despite that, based on these studies [49–51] we may conclude that both weight management and physical exercise could be beneficial to reduce nocturnal asthma prevalence and frequency in obese patients with asthma. Moreover, studies in children and adults with asthma show improvements in airway inflammation after physical training [41, 49, 72, 86, 87], that can lead to improved asthma control during the day and overnight. However, just 3 of the included studies evaluated the effects of physical exercise on inflammatory markers [41, 43, 49], and nocturnal symptoms were not the main outcome in these studies.

The mechanisms related to nocturnal worsening of asthma remain unclear. Nevertheless, the circadian changes in lung function as well as supine posture are known to contribute to the nocturnal worsening of asthma [16, 88]. Eight of the included studies evaluated the effects of physical training on daytime lung function [41–44, 46–49]. Seven of these studies [41, 43, 44, 46–49] as well as a previous systematic review [37] did not find changes in lung function related to physical exercise training. Although, the included studies did not evaluate overnight changes in lung function, it is unlikely that exercise training will cause overnight changes if it did not change daytime lung function. Another factor that may be associated with the nocturnal worsening of asthma is the overnight rostral fluid shift from the legs to the thorax which can increase fluid accumulation in the lower airway wall, narrow airway lumen, and worsen asthma symptoms [25, 26]. Previous studies in patients with sleep apnea have shown that physical training during the day can reduce overnight fluid shift out of the legs and improve sleep apnea severity [89] and snoring [90]. However, no study has evaluated the effect of exercise training on overnight rostral fluid shift and nocturnal asthma symptoms in patients with asthma.

Our systematic review has limitations that are important to address. First of all, we included a small number of studies. This was because many potentially relevant studies did not report nocturnal symptoms and subjective sleep quality data. Of 38 full text studies where authors were contacted, 71% of the authors could not be reached or did not have the requested data. Second, the majority of the included studies had a small number of participants and 50% of the studies had a high risk of bias for allocation, increasing the risk of type I error (false positive). Third, asthma medication was not reported in 3 studies [42, 49, 50] and 73% of the included studies did not report changes in asthma medications over the study period. Medication use may be an important potential confounder because it is related to both the ability to exercise and to asthma symptoms control, including nocturnal asthma symptoms. Fourth, our measurements of the frequency of nocturnal symptoms and subjective sleep quality were each based on a single subjective question. There is no standardized questionnaire to evaluate nocturnal symptoms in asthma. Sleep quality is impacted by a wide number of factors [91] but specific questionnaires or objective measurements such as polysomnography indices are not usually evaluated in asthma patients. Moreover, in our study we evaluated subjective sleep quality affected specifically by asthma symptoms. Lastly, the heterogeneous physical exercise routines and durations are also a limitation. We could not define minimum requirements for exercise regimens to optimize management of patients with nocturnal asthma based on our results. Additionally, the small number of randomized controlled trials (n = 5) and heterogeneity among them did not allow a meta-analysis. Despite the limited evidence, our review confirmed the high prevalence of nocturnal asthma in children and adults and the efficacy of physical exercise to improve nocturnal asthma control.

## Conclusions

In conclusion, our systematic review demonstrated that aerobic physical exercise may improve nocturnal asthma in children and adults by reducing the prevalence and frequency of nocturnal symptoms. The mechanisms by which physical activity improves nocturnal asthma remain unclear. For this reason, there is a need for more studies to evaluate the potential mechanisms of nocturnal worsening of asthma and how physical exercise might affect different asthma phenotypes. Such studies could lead to develop effective exercise routines as alternative or complementary treatments to improve asthma symptoms and quality of life in patients with nocturnal asthma.

## Supporting information

S1 Text MEDLINE search strategy. (DOCX)

S1 Table Summary table. (XLSX)

S2 Table Nocturnal asthma symptoms data. (XLSX)

S1 Checklist PRISMA checklist. (DOC)

## References

[1] Reddel HK, Taylor DR, Bateman ED, Boulet LP, Boushey HA, Busse WW, et al An official American Thoracic Society/European Respiratory Society statement: asthma control and exacerbations: standardizing endpoints for clinical asthma trials and clinical practice. Am J Respir Crit Care Med. 2009;180(1):59–99. 10.1164/rccm.200801-060ST 19535666

[2] Bousquet J, Knani J, Dhivert H, Richard A, Chicoye A, Ware JE Jr., et al Quality of life in asthma. I. Internal consistency and validity of the SF-36 questionnaire. Am J Respir Crit Care Med. 1994;149(2 Pt 1):371–5. 10.1164/ajrccm.149.2.8306032 8306032

[3] Bateman ED, Hurd SS, Barnes PJ, Bousquet J, Drazen JM, FitzGerald M, et al Global strategy for asthma management and prevention: GINA executive summary. The European respiratory journal. 2008;31(1):143–78. 10.1183/09031936.00138707 18166595

[4] To T, Stanojevic S, Moores G, Gershon AS, Bateman ED, Cruz AA, et al Global asthma prevalence in adults: findings from the cross-sectional world health survey. BMC Public Health. 2012;12:204 10.1186/1471-2458-12-204 22429515 PMC3353191

[5] Masoli M, Fabian D, Holt S, Beasley R, Global Initiative for Asthma P. The global burden of asthma: executive summary of the GINA Dissemination Committee report. Allergy. 2004;59(5):469–78. 10.1111/j.1398-9995.2004.00526.x 15080825

[6] Bahadori K, Doyle-Waters MM, Marra C, Lynd L, Alasaly K, Swiston J, et al Economic burden of asthma: a systematic review. BMC Pulm Med. 2009;9:24 10.1186/1471-2466-9-24 19454036 PMC2698859

[7] Braman SS. The global burden of asthma. Chest. 2006;130(1 Suppl):4S–12S. 10.1378/chest.130.1_suppl.4S 16840363

[8] Bellia V, Pistelli R, Filippazzo G, Cibella F, Scichilone N, Catalano F, et al Prevalence of nocturnal asthma in a general population sample: determinants and effect of aging. J Asthma. 2000;37(7):595–602. 11059527 10.3109/02770900009090815

[9] Diette GB, Markson L, Skinner EA, Nguyen TT, Algatt-Bergstrom P, Wu AW. Nocturnal asthma in children affects school attendance, school performance, and parents’ work attendance. Arch Pediatr Adolesc Med. 2000;154(9):923–8. 10980797 10.1001/archpedi.154.9.923

[10] Ballard RD. Sleep and medical disorders. Primary care. 2005;32(2):511–33. 10.1016/j.pop.2005.03.002 15935198

[11] Raherison C, Abouelfath A, Le Gros V, Taytard A, Molimard M. Underdiagnosis of nocturnal symptoms in asthma in general practice. The Journal of asthma: official journal of the Association for the Care of Asthma. 2006;43(3):199–202. 16754521 10.1080/02770900600566744

[12] Colice GL. Categorizing asthma severity and monitoring control of chronic asthma. Curr Opin Pulm Med. 2002;8(1):4–8. 11753117 10.1097/00063198-200201000-00002

[13] Colice GL. Categorizing asthma severity: an overview of national guidelines. Clin Med Res. 2004;2(3):155–63. 15931352 10.3121/cmr.2.3.155 PMC1069088

[14] Colice GL, Burgt JV, Song J, Stampone P, Thompson PJ. Categorizing asthma severity. Am J Respir Crit Care Med. 1999;160(6):1962–7. 10.1164/ajrccm.160.6.9902112 10588614

[15] Juniper EF, O’Byrne PM, Guyatt GH, Ferrie PJ, King DR. Development and validation of a questionnaire to measure asthma control. Eur Respir J. 1999;14(4):902–7. 10573240 10.1034/j.1399-3003.1999.14d29.x

[16] Calhoun WJ. Nocturnal asthma. Chest. 2003;123(3 Suppl):399S–405S. 12629002 10.1378/chest.123.3_suppl.399s

[17] Skloot GS. Nocturnal asthma: mechanisms and management. The Mount Sinai journal of medicine, New York. 2002;69(3):140–7. 12035073

[18] Stores G, Ellis AJ, Wiggs L, Crawford C, Thomson A. Sleep and psychological disturbance in nocturnal asthma. Arch Dis Child. 1998;78(5):413–9. 9659086 10.1136/adc.78.5.413 PMC1717552

[19] Fitzpatrick MF, Engleman H, Whyte KF, Deary IJ, Shapiro CM, Douglas NJ. Morbidity in nocturnal asthma: sleep quality and daytime cognitive performance. Thorax. 1991;46(8):569–73. 1926025 10.1136/thx.46.8.569 PMC463276

[20] Prasad B, Nyenhuis SM, Weaver TE. Obstructive sleep apnea and asthma: Associations and treatment implications. Sleep medicine reviews. 2014;18(2):165–71. 10.1016/j.smrv.2013.04.004 23890469

[21] Kraft M, Djukanovic R, Wilson S, Holgate ST, Martin RJ. Alveolar tissue inflammation in asthma. Am J Respir Crit Care Med. 1996;154(5):1505–10. 10.1164/ajrccm.154.5.8912772 8912772

[22] Hudgel DW, Devadatta P. Decrease in functional residual capacity during sleep in normal humans. J Appl Physiol Respir Environ Exerc Physiol. 1984;57(5):1319–22. 10.1152/jappl.1984.57.5.1319 6520027

[23] Muller N, Volgyesi G, Becker L, Bryan MH, Bryan AC. Diaphragmatic muscle tone. J Appl Physiol Respir Environ Exerc Physiol. 1979;47(2):279–84. 10.1152/jappl.1979.47.2.279 224022

[24] Muller NL, Francis PW, Gurwitz D, Levison H, Bryan AC. Mechanism of hemoglobin desaturation during rapid-eye-movement sleep in normal subjects and in patients with cystic fibrosis. Am Rev Respir Dis. 1980;121(3):463–9. 10.1164/arrd.1980.121.3.463 7416580

[25] Bhatawadekar SA, Inman MD, Fredberg JJ, Tarlo SM, Lyons OD, Keller G, et al Contribution of rostral fluid shift to intrathoracic airway narrowing in asthma. J Appl Physiol (1985). 2017;122(4):809–16. 28082337 10.1152/japplphysiol.00969.2016 PMC5504396

[26] Bhatawadekar SA, Keller G, Francisco CO, Inman MD, Fredberg JJ, Tarlo SM, et al Reduced Baseline Airway Caliber Relates to Larger Airway Sensitivity to Rostral Fluid Shift in Asthma. Frontiers in physiology. 2017;8(1012). 10.3389/fphys.2017.01012 PMC5733084 29311954

[27] Irvin CG, Pak J, Martin RJ. Airway-parenchyma uncoupling in nocturnal asthma. Am J Respir Crit Care Med. 2000;161(1):50–6. 10.1164/ajrccm.161.1.9804053 10619797

[28] Kraft M, Pak J, Martin RJ, Kaminsky D, Irvin CG. Distal lung dysfunction at night in nocturnal asthma. Am J Respir Crit Care Med. 2001;163(7):1551–6. 10.1164/ajrccm.163.7.2008013 11401872

[29] Kraft M, Hamid Q, Chrousos GP, Martin RJ, Leung DY. Decreased steroid responsiveness at night in nocturnal asthma. Is the macrophage responsible? Am J Respir Crit Care Med. 2001;163(5):1219–25. 10.1164/ajrccm.163.5.2002058 11316662

[30] Kraft M, Vianna E, Martin RJ, Leung DY. Nocturnal asthma is associated with reduced glucocorticoid receptor binding affinity and decreased steroid responsiveness at night. J Allergy Clin Immunol. 1999;103(1 Pt 1):66–71. 9893187 10.1016/s0091-6749(99)70527-0

[31] Desjardin JA, Sutarik JM, Suh BY, Ballard RD. Influence of sleep on pulmonary capillary volume in normal and asthmatic subjects. Am J Respir Crit Care Med. 1995;152(1):193–8. 10.1164/ajrccm.152.1.7599823 7599823

[32] O’Byrne PM, Bisgaard H, Godard PP, Pistolesi M, Palmqvist M, Zhu Y, et al Budesonide/formoterol combination therapy as both maintenance and reliever medication in asthma. Am J Respir Crit Care Med. 2005;171(2):129–36. 10.1164/rccm.200407-884OC 15502112

[33] Aronow WS, Fleg JL, Pepine CJ, Artinian NT, Bakris G, Brown AS, et al ACCF/AHA 2011 expert consensus document on hypertension in the elderly: a report of the American College of Cardiology Foundation Task Force on Clinical Expert Consensus Documents. Circulation. 2011;123(21):2434–506. 10.1161/CIR.0b013e31821daaf6 21518977

[34] Cotman CW, Berchtold NC, Christie LA. Exercise builds brain health: key roles of growth factor cascades and inflammation. Trends Neurosci. 2007;30(9):464–72. 10.1016/j.tins.2007.06.011 17765329

[35] Pastva A, Estell K, Schoeb TR, Atkinson TP, Schwiebert LM. Aerobic exercise attenuates airway inflammatory responses in a mouse model of atopic asthma. J Immunol. 2004;172(7):4520–6. 15034069 10.4049/jimmunol.172.7.4520 PMC2892102

[36] de Freitas Dantas Gomes EL, Costa D. Evaluation of functional, autonomic and inflammatory outcomes in children with asthma. World J Clin Cases. 2015;3(3):301–9. doi: 10.12998/wjcc.v3.i3.301 25789303 10.12998/wjcc.v3.i3.301 PMC4360502

[37] Carson KV, Chandratilleke MG, Picot J, Brinn MP, Esterman AJ, Smith BJ. Physical training for asthma. Cochrane Database Syst Rev. 2013(9):CD001116 10.1002/14651858.CD001116.pub4 24085631 PMC11930393

[38] Eichenberger PA, Diener SN, Kofmehl R, Spengler CM. Effects of exercise training on airway hyperreactivity in asthma: a systematic review and meta-analysis. Sports Med. 2013;43(11):1157–70. 10.1007/s40279-013-0077-2 23846823

[39] Higgins J, Green S. Cochrane handbook for systematic reviews of interventions: John Wiley & Sons; 2011.

[40] Moher D, Liberati A, Tetzlaff J, Altman DG, Group P. Preferred reporting items for systematic reviews and meta-analyses: the PRISMA statement. PLoS Med. 2009;6(7):e1000097 10.1371/journal.pmed.1000097 19621072 PMC2707599

[41] Gomes ELFD, Carvalho CRF, Peixoto-Souza FS, Teixeira-Carvalho EF, Mendonca JFB, Stirbulov R, et al Active video game exercise training improves the clinical control of asthma in children: Randomized controlled trial. PLoS ONE. 2015;10 (8)(0135433). 10.1371/journal.pone.0135433 PMC4547724 26301706

[42] Haines MS, Kim DH. A Study of the Effects of Physical Activity on Asthmatic Symptoms and Obesity Risk in Elementary School-Aged Children. American Journal of Health Education. 2013;44(3):156–61.

[43] Moreira A, Delgado L, Haahtela T, Fonseca J, Moreira P, Lopes C, et al Physical training does not increase allergic inflammation in asthmatic children. Eur Respir J. 2008;32(6):1570–5. 10.1183/09031936.00171707 18684843

[44] Weisgerber MC, Guill M, Weisgerber JM, Butler H. Benefits of swimming in asthma: effect of a session of swimming lessons on symptoms and PFTs with review of the literature. J Asthma. 2003;40(5):453–64. 14529095 10.1081/jas-120018706

[45] Westergren T, Fegran L, Nilsen T, Haraldstad K, Kittang OB, Berntsen S. Active play exercise intervention in children with asthma: a PILOT STUDY. BMJ Open. 2016;6(1):e009721 10.1136/bmjopen-2015-009721 26733570 PMC4716232

[46] Dogra S, Jamnik V, Baker J. Self-directed exercise improves perceived measures of health in adults with partly controlled asthma. J Asthma. 2010;47(9):972–7. 10.1080/02770903.2010.508857 20868317

[47] Dogra S, Kuk JL, Baker J, Jamnik V. Exercise is associated with improved asthma control in adults. Eur Respir J. 2011;37(2):318–23. 10.1183/09031936.00182209 20530042

[48] Hallstrand TS, Bates PW, Schoene RB. Aerobic conditioning in mild asthma decreases the hyperpnea of exercise and improves exercise and ventilatory capacity. Chest. 2000;118(5):1460–9. 11083702 10.1378/chest.118.5.1460

[49] Scott HA, Gibson PG, Garg ML, Pretto JJ, Morgan PJ, Callister R, et al Dietary restriction and exercise improve airway inflammation and clinical outcomes in overweight and obese asthma: a randomized trial. Clin Exp Allergy. 2013;43(1):36–49. 10.1111/cea.12004 23278879

[50] Scott HA, Gibson PG, Garg ML, Pretto JJ, Morgan PJ, Callister R, et al Determinants of weight loss success utilizing a meal replacement plan and/or exercise, in overweight and obese adults with asthma. Respirology. 2015;20(2):243–50. 10.1111/resp.12423 25366866

[51] Turk Y, van Huisstede A, Franssen FME, Hiemstra PS, Rudolphus A, Taube C, et al Effect of an Outpatient Pulmonary Rehabilitation Program on Exercise Tolerance and Asthma Control in Obese Asthma Patients. J Cardiopulm Rehabil Prev. 2017;37(3):214–22. 10.1097/HCR.0000000000000249 28448379

[52] Juniper EF, Gruffydd-Jones K, Ward S, Svensson K. Asthma Control Questionnaire in children: validation, measurement properties, interpretation. Eur Respir J. 2010;36(6):1410–6. 10.1183/09031936.00117509 20530041

[53] Juniper EF, Bousquet J, Abetz L, Bateman ED, Committee G. Identifying ‘well-controlled’ and ‘not well-controlled’ asthma using the Asthma Control Questionnaire. Respir Med. 2006;100(4):616–21. 10.1016/j.rmed.2005.08.012 16226443

[54] Juniper EF, Svensson K, Mork AC, Stahl E. Measurement properties and interpretation of three shortened versions of the asthma control questionnaire. Respiratory medicine. 2005;99(5):553–8. 10.1016/j.rmed.2004.10.008 15823451

[55] Juniper EF, Guyatt GH, Cox FM, Ferrie PJ, King DR. Development and validation of the Mini Asthma Quality of Life Questionnaire. Eur Respir J. 1999;14(1):32–8. 10489826 10.1034/j.1399-3003.1999.14a08.x

[56] Juniper EF, Guyatt GH, Feeny DH, Ferrie PJ, Griffith LE, Townsend M. Measuring quality of life in the parents of children with asthma. Qual Life Res. 1996;5(1):27–34. 8901364 10.1007/BF00435966

[57] Juniper EF, Guyatt GH, Willan A, Griffith LE. Determining a minimal important change in a disease-specific Quality of Life Questionnaire. J Clin Epidemiol. 1994;47(1):81–7. 8283197 10.1016/0895-4356(94)90036-1

[58] Andrade LB, Britto MC, Lucena-Silva N, Gomes RG, Figueroa JN. The efficacy of aerobic training in improving the inflammatory component of asthmatic children. Randomized trial. Respir Med. 2014;108(10):1438–45. 10.1016/j.rmed.2014.07.009 25231109

[59] Basaran S, Guler-Uysal F, Ergen N, Seydaoglu G, Bingol-Karakoc G, Ufuk Altintas D. Effects of physical exercise on quality of life, exercise capacity and pulmonary function in children with asthma. J Rehabil Med. 2006;38(2):130–5. 10.1080/16501970500476142 16546771

[60] Bingol Karakoc G, Yilmaz M, Sur S, Ufuk Altintas D, Sarpel T, Guneter Kendirli S. The effects of daily pulmonary rehabilitation program at home on childhood asthma. Allergol Immunopathol (Madr). 2000;28(1):12–4. 10757852

[61] Boyd A, Yang CT, Estell K, Ms CT, Gerald LB, Dransfield M, et al Feasibility of exercising adults with asthma: a randomized pilot study. Allergy Asthma Clin Immunol. 2012;8(1):13 10.1186/1710-1492-8-13 22863207 PMC3511803

[62] Cox NJM, Hendricks JC, Binkhorst RA, Van Herwaarden CLA. A pulmonary rehabilitation program for patients with asthma and mild chronic obstructive pulmonary diseases (COPD). Lung. 1993;171(4):235–44. 8341090 10.1007/BF00203723

[63] Fanelli A, Cabral AL, Neder JA, Martins MA, Carvalho CR. Exercise training on disease control and quality of life in asthmatic children. Med Sci Sports Exerc. 2007;39(9):1474–80. 10.1249/mss.0b013e3180d099ad 17805077

[64] Fitch KD, Morton AR, Blanksby BA. Effects of swimming training on children with asthma. Arch Dis Child. 1976;51(3):190–4. 782376 10.1136/adc.51.3.190 PMC1545918

[65] Foglio K, Bianchi L, Bruletti G, Battista L, Pagani M, Ambrosino N. Long-term effectiveness of pulmonary rehabilitation in patients with chronic airway obstruction. Eur Respir J. 1999;13(1):125–32. 10836336 10.1183/09031936.99.13112599

[66] Franca-Pinto A, Mendes FA, de Carvalho-Pinto RM, Agondi RC, Cukier A, Stelmach R, et al Aerobic training decreases bronchial hyperresponsiveness and systemic inflammation in patients with moderate or severe asthma: a randomised controlled trial. Thorax. 2015;70(8):732–9. 10.1136/thoraxjnl-2014-206070 26063507

[67] Freitas PD, Ferreira PG, Silva AG, Stelmach R, Carvalho-Pinto RM, Fernandes FL, et al The Role of Exercise in a Weight-Loss Program on Clinical Control in Obese Adults with Asthma. A Randomized Controlled Trial. Am J Respir Crit Care Med. 2017;195(1):32–42. 10.1164/rccm.201603-0446OC 27744739

[68] Holzer FJ, Schnall R, Landau LI. The effect of a home exercise programme in children with cystic fibrosis and asthma. Aust Paediatr J. 1984;20(4):297–301. 6529386 10.1111/j.1440-1754.1984.tb00098.x

[69] Latorre-Roman PA, Navarro-Martinez AV, Garcia-Pinillos F. The effectiveness of an indoor intermittent training program for improving lung function, physical capacity, body composition and quality of life in children with asthma. J Asthma. 2014;51(5):544–51. 10.3109/02770903.2014.888573 24471516

[70] Ma J, Strub P, Xiao L, Lavori PW, Camargo CA Jr., Wilson SR, et al Behavioral weight loss and physical activity intervention in obese adults with asthma. A randomized trial. Ann Am Thorac Soc. 2015;12(1):1–11. 10.1513/AnnalsATS.201406-271OC 25496399 PMC4342805

[71] Majewski M, Dabrowska G, Pawik M, Rozek K. Evaluation of a Home-Based Pulmonary Rehabilitation Program for Older Females Suffering from Bronchial Asthma. Adv. 2015;24(6):1079–83. 10.17219/acem/31679 26771982

[72] Mendes FA, Almeida FM, Cukier A, Stelmach R, Jacob-Filho W, Martins MA, et al Effects of aerobic training on airway inflammation in asthmatic patients. Med Sci Sports Exerc. 2011;43(2):197–203. 10.1249/MSS.0b013e3181ed0ea3 20581719

[73] Mendes FA, Goncalves RC, Nunes MP, Saraiva-Romanholo BM, Cukier A, Stelmach R, et al Effects of aerobic training on psychosocial morbidity and symptoms in patients with asthma: a randomized clinical trial. Chest. 2010;138(2):331–7. 10.1378/chest.09-2389 20363839

[74] Neder JA, Nery LE, Silva AC, Cabral ALB, Fernandes ALG. Short term effects of aerobic training in the clinical management of moderate to severe asthma in children. Thorax. 1999;54(3):202–6. 10325894 10.1136/thx.54.3.202 PMC1745434

[75] Gonçalves RC, Nunes MPT, Cukier A, Stelmach R, Martins MA, Carvalho CRF. Effects of an aerobic physical training program on psychosocial characteristics, quality-of-life, symptoms and exhaled nitric oxide in individuals with moderate or severe persistent asthma. Braz J Phys Ther. 2008;12(2):127–35.

[76] Refaat A, Gawish M. Effect of physical training on health-related quality of life in patients with moderate and severe asthma. Egypt J Chest Dis Tuberc. 2015;64(4):761–6.

[77] Robinson DM, Egglestone DM, Hill PM, Rea HH, Richards GN, Robinson SM. Effects of a physical conditioning programme on asthmatic patients. N Z Med J. 1992;105(937):253–6. 1620508

[78] Schnall R, Ford P, Gillam I, Landau L. Swimming and dry land exercises in children with asthma. Aust Paediatr J. 1982;18(1):23–7. 7103876 10.1111/j.1440-1754.1982.tb01973.x

[79] Szentagothai K, Gyene I, Szocska M, Osvath P. Physical exercise program for children with bronchial asthma. Pediatr Pulmonol. 1987;3(3):166–72. 3615039 10.1002/ppul.1950030310

[80] Turner S, Eastwood P, Cook A, Jenkins S. Improvements in symptoms and quality of life following exercise training in older adults with moderate/severe persistent asthma. Respiration. 2011;81(4):302–10. 10.1159/000315142 20501982

[81] Wang JS, Hung WP. The effects of a swimming intervention for children with asthma. Respirology. 2009;14(6):838–42. 10.1111/j.1440-1843.2009.01567.x 19703065

[82] Weisgerber M, Webber K, Meurer J, Danduran M, Berger S, Flores G. Moderate and vigorous exercise programs in children with asthma: safety, parental satisfaction, and asthma outcomes. Pediatr Pulmonol. 2008;43(12):1175–82. 10.1002/ppul.20895 19003892

[83] Zolaktaf V, Ghasemi GA, Sadeghi M. Effects of exercise rehab on male asthmatic patients: aerobic verses rebound training. Int J Prev Med. 2013;4(Suppl 1):S126–32. 23717762 PMC3665018

[84] Barnes PJ. Inflammatory mechanisms and nocturnal asthma. Am J Med. 1988;85(1B):64–70. 3041827 10.1016/0002-9343(88)90245-8

[85] Martin RJ, Cicutto LC, Smith HR, Ballard RD, Szefler SJ. Airways inflammation in nocturnal asthma. Am Rev Respir Dis. 1991;143(2):351–7. 10.1164/ajrccm/143.2.351 1990952

[86] Boyd A, Estell K, Dransfield M, Schwiebert L. The effect of aerobic exercise on asthma-related responses in adults. J Allergy Clin Immunol. 2011;127(2):AB223.

[87] Matsumoto I, Araki H, Tsuda K, Odajima H, Nishima S, Higaki Y, et al Effects of swimming training on aerobic capacity and exercise induced bronchoconstriction in children with bronchial asthma. Thorax. 1999;54(3):196–201. 10325893 10.1136/thx.54.3.196 PMC1745437

[88] Bohadana AB, Hannhart B, Teculescu DB. Nocturnal worsening of asthma and sleep-disordered breathing. J Asthma. 2002;39(2):85–100. 11990234 10.1081/jas-120002190

[89] Mendelson M, Lyons OD, Yadollahi A, Inami T, Oh P, Bradley TD. Effects of exercise training on sleep apnoea in patients with coronary artery disease: a randomised trial. Eur Respir J. 2016;48(1):142–50. 10.1183/13993003.01897-2015 27076578

[90] Singh B, Yadollahi A, Lyons O, Alshaer H, Bradley TD. The effect of sitting and calf activity on leg fluid and snoring. Respir Physiol Neurobiol. 2017;240:1–7. 10.1016/j.resp.2017.02.008 28214605

[91] Buysse DJ, Reynolds CF 3rd, Monk TH, Berman SR, Kupfer DJ. The Pittsburgh Sleep Quality Index: a new instrument for psychiatric practice and research. Psychiatry Res. 1989;28(2):193–213. 2748771 10.1016/0165-1781(89)90047-4

