# Supplementary material for: Effects of physical exercise training on nocturnal symptoms in asthma: Systematic review
Source: PLoS One. 2018 Oct 22;13(10):e0204953. doi: 10.1371/journal.pone.0204953 (PMC6197640; doi:10.1371/journal.pone.0204953)
Supplement: S1 Text — (DOCX) [file pone.0204953.s001.docx]

**Supplementary material:** MEDLINE Search strategy

Database: Ovid MEDLINE(R) Epub Ahead of Print, In-Process & Other Non-Indexed Citations, Ovid MEDLINE(R) Daily and Ovid MEDLINE(R)

Search Strategy:

1 exp asthma/

2 asthma*.mp.

3 1 or 2

4 exp Exercise/
5 exp Exercise Therapy/

6 exp Exercise Movement Techniques/

7 Physical Fitness/

8 exp "Physical Education and Training"/

9 (exercise* or exercising).tw,kw.

10 (gi gong or gigong).tw,kw.

11 ((tai adj ji) or ((tai or thai) adj chi) or taiji or taijiquan or taichi).tw,kw.

12 walking.tw,kw.

13 yoga.tw,kw.

14 (physical adj (fitness or condition* or education or training or mobility or activit* or exertion or effort)).tw,kw.

15 gymnastic*1.tw,kw.

16 calisthenics.tw,kw.

17 aerobic*1.tw,kw.

18 danc*.tw,kw.

19 (jumping or hopping).tw,kw.

20 (running or jogging).tw,kw.

21 ambulat*.tw,kw.

22 muscle strengthening.tw,kw.

23 ((strength or resistance) adj training).tw,kw.

24 ((weight*1 adj2 lifting) or weightlifting or power lifting or weight training).tw,kw.

25 pilates.tw,kw.

26 stretching.tw,kw.

27 plyometric*.tw,kw.

28 (cardio* adj conditioning).tw,kw.

29 ((physical or motion or movement or recreation or activity) adj therap*).tw,kw.

30 isometric training.tw,kw.

31 climbing.tw,kw.

32 cycling.tw,kw.

33 (swim or swimming).tw,kw.

34 (training adj (course* or program*)).tw,kw.

35 kinesi?therap*.tw,kw.

36 exp Sports/

37 or/4-36

38 3 and 37

39 exp clinical trial/

40 exp Clinical Trials as Topic/

41 Multicenter Studies as Topic/

42 double-blind method/ or single-blind method/

43 random*.tw,kw.

44 (controlled adj3 trial*).tw,kw.

45 (clinical adj3 trial*).tw,kw.

46 placebo.tw,kw.

47 trial.ti,ab.

48 groups.ab.

49 or/39-48

50 38 and 49

51 50 not (exp animals/ not exp humans/)

52 limit 51 to english language
